# Study results from journals with a higher impact factor are closer to “truth”: a meta-epidemiological study

**DOI:** 10.1186/s13643-023-02167-8

**Published:** 2023-01-18

**Authors:** Andreas Heidenreich, Nora Eisemann, Alexander Katalinic, Joachim Hübner

**Affiliations:** Institute of Social Medicine and Epidemiology, University of Luebeck, Ratzeburger Allee 160, 23538 Luebeck, Germany

**Keywords:** Meta-epidemiology, Cochrane, Journal impact factor, Bibliometrics

## Abstract

**Background:**

Scientists, physicians, and the general public legitimately expect scholarly publications to give true answers to study questions raised. We investigated whether findings from studies published in journals with higher *Journal Impact Factors* (JIFs) are closer to truth than findings from studies in less-cited journals via a meta-epidemiological approach.

**Methods:**

We screened intervention reviews from the Cochrane Database of Systematic Reviews (CDSR) and sought well-appraised meta-analyses. We used the individual RCT study estimates’ relative deviation from the pooled effect estimate as a proxy for the deviation of the study results from the truth. The effect of the JIF on the relative deviation was estimated with linear regression and with local polynomial regression, both with adjustment for the relative size of studies. Several sensitivity analyses for various sub-group analyses and for alternative impact metrics were conducted.

**Results:**

In 2459 results from 446 meta-analyses, results with a higher JIF were on average closer to “truth” than the results with a lower JIF. The relative deviation decreased on average by −0.023 per JIF (95% CI −0.32 to −0.21). A decrease was consistently found in all sensitivity analyses.

**Conclusions:**

Our results indicate that study results published in higher-impact journals are on average closer to truth. However, the JIF is only one weak and impractical indicator among many that determine a studies’ accuracy.

## Key messages

- The deviation of study results to the truth was measured using pooled estimates of good-quality meta-analyses as proxies for truth.
- RCT study results published in higher-impact journals lie closer to “true” values than results published in lower-impact journals.
- As the association found is weak, the Journal Impact Factor is of limited practical value in predicting the trueness of a study result.

“In 1955, it did not occur to me that “impact” would one day become so controversial. Like nuclear energy, the impact factor is a mixed blessing. I expected it to be used constructively while recognising that in the wrong hands it might be abused.” – Eugene Garfield, 2005

## Introduction

Academic publications are an important and much-noticed output of scientific work. The general public, policymakers, the scientific community, and physicians legitimately expect publications to give true answers to their study questions. However, there is increasing concern that the majority of published research findings are false [1]. In a constantly growing flood of publications, preferential trust is put in journals with a high *Journal Impact Factor*(JIF) [2]. Despite extensive criticism relating to its manipulability and widespread misuse, e.g., as a measure of the scientific performance of researchers and institutions [3–5], existing research supports the notion of a relation between the impact factor of a journal and the quality of research published therein. Concerning clinical research, it has been shown that randomized controlled trials (RCTs) published in higher-impact journals are less prone to risk of bias compared with those published in lower-impact journals in their reported design, conduct, and analysis [6–8]. Furthermore, lower methodological quality is associated with larger, commonly beneficial effects [9, 10]. Taking these findings together, one could come to the conclusion that studies from lower impact journals tend to overestimate the true effect of an intervention. Contradicting this assumption, it has been found that trials published in the highest-impact journals show more favorable effects for experimental interventions than trials from other journals. As this difference has not been found for large trials, the exceeding effect size of small trials in high-impact journals provoked caveats regarding their reliability [11].

To our knowledge, the direct association between the impact factor of a journal in which a study is published and the extent to which the study results tell us the truth has not been investigated to date. One possible reason for this is the obvious difficulty of determining the truth in empirical science. This is all the more the case when it is not a question of truth as a categorical statement, but of the magnitude of a true value. In medical science, this epistemological challenge can be met in a practically suitable manner. The idea is that the pooled effect estimates from distinctly reliable meta-analyses represent true values sufficiently well to serve as a proxy measure of truth. Cochrane reviews provide pertinent meta-analyses. Compared with other systematic reviews, Cochrane reviews use more rigorous methods [12–14], laid down in a comprehensive, periodically updated handbook [15]. An integral part of these methods is to assess the quality of the underlying body of evidence (sometimes referred to as “certainty of evidence”) on a four-level scale. This results in a transparently structured judgment about the likelihood of trueness of the pooled effect estimate. The upper two levels (“high” and “moderate” quality) indicate that the authors are “very” or “moderately” confident that the true effect “lies close” (or “is likely to be close”) to the estimate of the effect.

Following this assessment, we postulated that effect estimates from Cochrane meta-analyses based on evidence of “high” or “moderate” quality are sufficient proxies of true values. To distinguish between the actual, unknown truth and a proxy estimate of the truth, we refer to the latter as “truth” (in quotation marks). We examined whether the results of intervention studies published in higher-impact journals lie closer to the “truth” compared with those published in lower-impact journals. To better understand the hypothesised relationship, we fitted non-linear regression models, adjusted for confounders, and conducted sensitivity analyses for various subgroups and for alternative metrics of impact.

## Methods

### Selection of eligible cochrane reviews and meta-analyses

We prospectively screened all intervention reviews published in the Cochrane Database of Systematic Reviews (CDSR) from July 2018 to January 2019 for meta-analyses. We restricted the selection to meta-analyses generating evidence of “moderate” or “high” quality. In this respect, we adopted the review author’s assessment based on the GRADE framework. Therein, “moderate” or “high” quality of evidence represents the upper two of four levels (the others being “low” and “very low”). These judgments imply that the review authors consider the pooled effect estimates close to the true values with “moderate” or “high” certainty, respectively [16]. We decided against a limitation to meta-analysis with “high”-quality evidence since only few meta-analyses are rated as such. In a pre-study, we found that of 986 examined meta-analyses published in 59 Cochrane intervention reviews, only 26 (2.6%) were of “high” quality of evidence and the proportion of “moderate”-quality meta-analyses was also small (7.7%). Thus, we considered pooled effect estimates of both “high” and “moderate” certainty to be suitable proxies for truth.

In case a review contained at least one meta-analysis of moderate or high-quality evidence, we obtained the accompanying data file from the Cochrane website and extracted the corresponding data via Cochrane’s *Review Manager*software, version 5.3 [17]. We entered the extracted data and identifying information on the meta-analysis into our study database.

### Matching study results with publication data

For each study result included in eligible meta-analyses, we identified the source publication. When more than one publication was referenced, we screened the articles’ abstracts and full texts to identify the correct publication reliably, as not in every case the designated primary publication was the source of all data used in meta-analyses.

### Journal’s impact

For each publication included in eligible meta-analyses, we obtained information on the publishing journal’s JIF of the publication year from the Journal Citations Report (Clarivate Analytics). We preferred this indicator to others (e.g., SCImago Journal Rank (SJR), Eigenfactor (EF), and H factor) for primary analysis, as it represents the de-facto standard of citation metrics in science and is most likely known outside the scientific community. Information on the JIF was available from 1997 to 2018. We excluded results reported in older publications from the analysis.

As the average reference list in the medical sciences and other fields became lengthier over time, JIFs are subject to inflation [18, 19]. We did not consider this increase as a real accretion in impact on the process of science. We, therefore, adjusted the JIF for publication date by calculating the ratio of the actual JIF and the mean value of all journals’ JIFs in our study sample of a given year. The same was done for SJR and EF. The H-Factor, calculated by the SJR’s publisher Scimago Lab on the basis of the Scopus database, was only available at the time of data entry.

### Operationalization of the outcome measure

The purpose of most scientific medical studies is to estimate some unknown true parameter value based on data from a study sample. The study estimates can deviate from the true value because of systematic error (bias) and random error (chance; lack of precision). Obviously, it was impossible for us to measure the true values, and a proxy variable for truth had to be employed. Meta-analyses combine the results of multiple studies to derive a pooled estimate that approximates the true value more accurately. In the absence of better alternatives and aware of the limitations of this choice, we defined the pooled effect estimate of the respective meta-analysis as “truth.” The outcome of our analysis, the closeness of a single study’s point estimate to the “truth,” was operationalised as the *relative deviation*. The point estimates were centred around their respective “true” value and then standardized with the average distance of the point estimates to the pooled effect estimate of studies in the meta-analysis. Risk ratio (RR), odds ratio (OR), and hazard ratio (HR) were transformed from their multiplicative scale to an additive scale to enable a comparison of different effect measures (RR and OR for dichotomous outcomes, HR for time-dependent outcomes, mean difference in various units of measurement and standardised mean difference for continuous outcomes). As it does not matter if a deviation from the truth is an over- or underestimation, negative signs were removed from the deviation measures.

In summary, a relative deviation of 1 means that a point estimate’s deviation from its pooled effect estimate corresponds to the average of all deviations of the point estimates of a given meta-analysis. When a meta-analysis reports a treatment vs. control pooled effect estimate of RR = 0.70 while one of the meta-analysis’ studies reports an RR of 0.80, that study has a distance to its pooled effect of |log(0.70)–log(0.80)|= 0.13. When the studies in the meta-analysis scatter around the pooled effect estimate with an average distance of 0.10, our example displays a relative deviation of 0.13/10 = 1.30; a value of > 1 indicating an above-average deviation.

Our approach becomes questionable when a single study dominates a meta-analysis. The pooled effect estimate is calculated as a weighted average of the intervention effects estimated in the individual studies [20]. The more a pooled effect estimate is influenced by a particular study, the less it may serve as an appropriate benchmark for the accuracy of this very study. This is obvious when a meta-analysis comprises only one individual study. According to our definition, this study cannot fail to meet the “truth.” Therefore, we considered all individual studies with a weight of 50% or more as non-informative regarding our research question and excluded them from further analysis. We also excluded study results with no point estimates, e.g., results with no events in either study arm for dichotomous outcome measures or missing standard deviations in the case of continuous outcome measures [20].

### Covariates

To control for confounders, we considered variables that we expected to be (causal) predecessors of both the JIF and the relative deviation.

First, we suspected that larger studies with a correspondingly smaller variance of the effect measure are more likely to be published in higher impact journals than smaller studies with larger variance. As smaller variance translates to bigger weight in meta-analyses, the suspected association would result in the observation that studies from higher impact journals show systematically smaller relative deviations. As the risk of confounding has been mitigated but not eliminated by the exclusion of studies with a weight of 50% or more, we considered the study weight as a confounder.

Second, we assumed that a higher methodological quality of studies (in terms of compliance with the methodological requirements of a journal and thus a lower risk of bias) reduces the relative deviation and that studies with a lower risk of bias tend to be published in journals with a higher JIF than studies with a higher risk of bias. On the other hand, the range in risk of bias of studies that a journal usually accepts for publication can be seen as a characteristic of a journal. Then, the effect of the JIF on the relative deviation is partly due to the risk of bias acceptable to that journal. We adopted the latter view for analysis and quantified the proportion of explanatory power that is due to the risk of bias.

For every study included in Cochrane reviews, the results of a detailed and mostly standardized risk of bias assessment conducted by the review authors are reported [20]. Usually, seven key items are assessed for each study (“random sequence generation,” “allocation concealment,” “selective reporting,” “blinding of participants and personnel,” “blinding of outcome assessment,” “incomplete outcome data”, and “other”). We transformed the qualitative verdicts (“low risk of bias,” “unclear risk of bias,” and “high risk of bias”) for each key item to numerical values (0.0, 0.5, and 1.0, respectively) and calculated the mean value. The result is an aggregated risk of bias between “0” (low risk of bias in every item) and “1” (high risk of bias in every item). As this approach truncates the qualitative dimensions of the risk of bias assessment, it is not suitable for comprehensive critical appraisals of an RCT. Here, we pursue a general statistical relationship, and a loss of qualitative information appears acceptable.

### Statistical analysis

The mean of the relative deviation, i.e., the weighted deviation of a study estimate published in that journal to the “truth” as defined above, was compared between study results from journals with an associated JIF and those without a JIF by using a Welsh *t* test.

Focusing on publications with a JIF, we assessed the effect of the JIF on the relative deviation by fitting a linear regression. The regression was adjusted for the assumed confounder, “study weight,” and weighted with the JIF to account for heteroscedasticity of the model residuals. Then, visual model diagnostics (mean of 0 and homoscedasticity across all fitted values and the predictor) indicated at most small deviations from the modelling assumptions. The assumption of normality of residuals was not met, but the central limit theorem came into force due to the large sample size. From a theoretical point of view, the observations were clustered within their respective meta-analyses and were consequently not independent. However, a mixed effects model with random intercept and slope for each meta-analysis found a variance of 0 for both random intercept and slope, indicating that a fixed effects regression is sufficient. Three extreme values (with relative deviations of 11.6, 6.4, and 6.3, JIFs 0.22, 1.0, and 1.0) were excluded for more robust results and a better graphical representation.

As the linearity of the relationship between JIF and relative deviation is a quite strong assumption, we additionally allowed for a relationship without a pre-specified functional form by fitting a local polynomial regression (R function stats::loess). Again, the model was adjusted for study weight in the meta-analysis and observations were weighted by their JIF.

The regression curves of both models were plotted together with their 95% confidence bands while fixing the value for the adjustment variable “study weight” at its median value of 7.41%.

Subgroup analyses were conducted for (1) “high” vs. “moderate” quality of evidence, (2) open access vs. traditional publishing, (3) large (ten or more included studies) vs. small meta-analyses, (4) meta-analyses using random effects vs. fixed effects for the included studies, (5) low vs. moderate study weight (median split at 7.41%), (6) low vs. high risk of bias (median split at 0.21), and (7) study results using RR vs. OR vs. mean differences as effect measures. In all cases, the linear regression and the local polynomial regression are shown, both adjusted and weighted as in the main analysis.

In addition to the JIF, the scientific journal ranking (SJR; adjusted for inflation), eigenfactor (EF; adjusted for inflation), and the H-factor were analyzed. Fourteen observations (EF ranging between 43 and 69) were excluded because they exhibited a strong leverage effect. Again, linear as well as local polynomial regressions were fitted for each outcome. The regressions were weighted with the respective measure of impact to control heteroscedasticity of residuals.

The proportion of explanatory power of the JIF due to the risk of bias was calculated as the proportion of the increase in multiple *R*^2^ when adding JIF as predictor to the basic adjusted and weighted model, that could be attributed to the risk of bias predictor: ((*R*^2^_JIF model_—*R*^2^_basic model_) + (*R*^2^_ROB model_—*R*^2^_basic model_)—(*R*^2^_JIF and ROB model_—*R*^2^_basic model_))/(*R*^2^_JIF model_—*R*^2^_basic model_).

We further assessed if study results from small studies published in high-impact journals are especially far from “truth” by adding an interaction term between JIF and study weight to the main linear regression.

All statistical analysis was performed with the *R *software in version 4.1.3 [21].

## Results

**Fig. 1:**
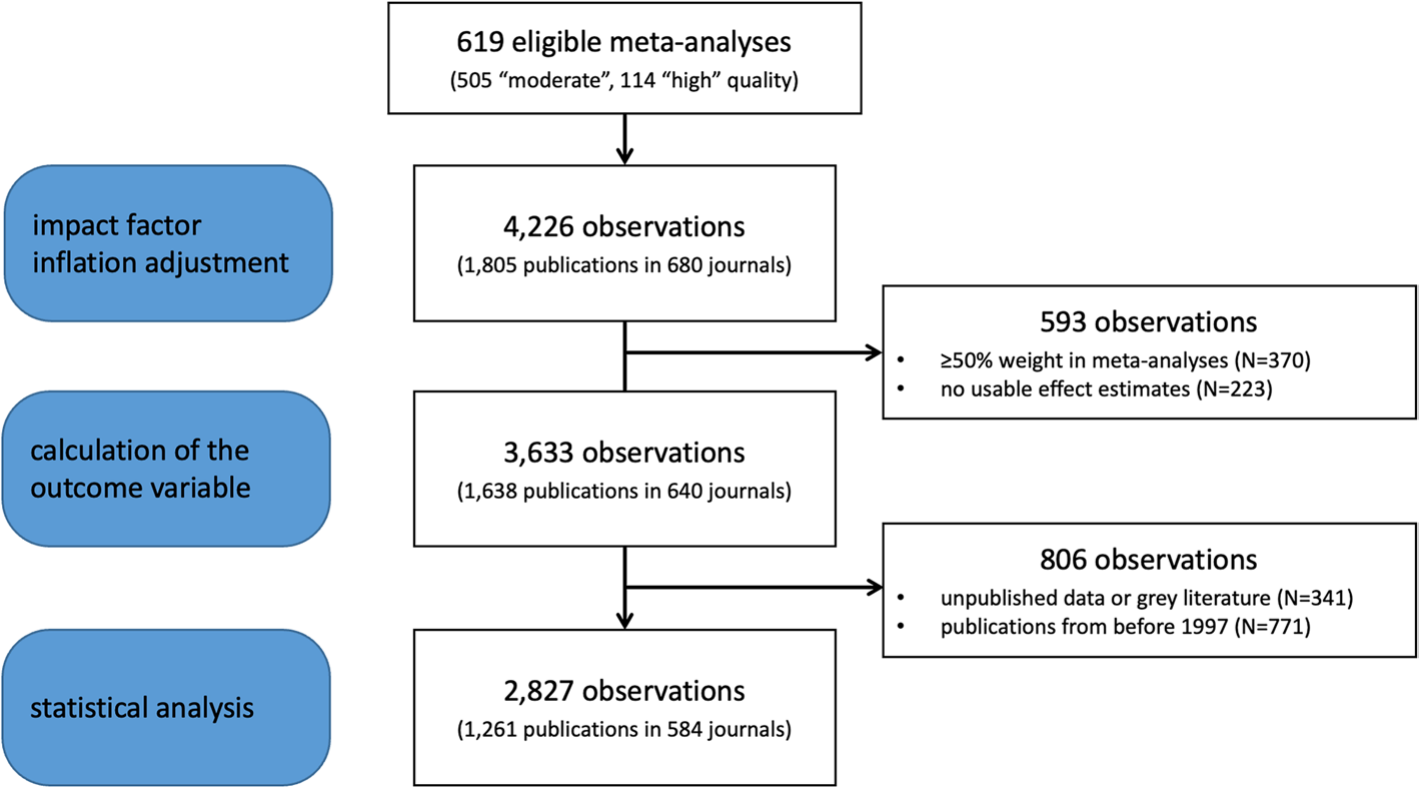
Inclusion and exclusion criteria at various steps of analysis

We obtained 4226 study results from 619 eligible Cochrane meta-analyses and presented in 148 reviews (Fig. 1). The application of exclusion criteria resulted in a sample of 2827 study results from 455 meta-analyses, of which nearly 20% were of “high” and 80% of “moderate” quality of evidence (Table 1). All meta-analyses included only RCTs.

**Table 1:** Description of the 2827 included study results and their 455 meta-analyses

	**Total**		**Journals with JIF**	
	**Study results** **(*N* = 2827)**	**Meta-analyses** **(*N* = 455)**	**Study results** **(*N* = 2459)**	**Meta-analyses** **(*N* = 446)**
**Quality of evidence**				
High, *N* (%)	652 (23.1)	87 (19.1)	607 (24.7)	86 (19.3)
Moderate, *N* (%)	2175 (76.9)	368 (80.9)	1852 (75.3)	360 (80.7)
**Open access**				
Yes, *N* (%)	239 (8.5)	NA	190 (7.7)	NA
**Size of meta-analysis**				
Ten or more studies, *N* (%)	1545 (54.7)	119 (26.2)	1330 (54.1)	118 (26.5)
**Pooling method**				
Fixed effects model, *N* (%)	1003 (35.5)	188 (41.3)	864 (35.1)	186 (41.7)
Random effects model, *N* (%)	1824 (64.5)	267 (58.7)	1595 (64.9)	260 (58.3)
**Study weight (in %)**				
Median (1. to 3. Quartile)	7.1 (2.8 to 16.6)	NA	7.4 (2.9 to 16.8)	NA
Range	< 0.05 to < 50	NA	< 0.05 to < 50	NA
**Risk of bias**				
Median (1. to 3. Quartile)	0.21 (0.12 to 0.36)	NA	0.21 (0.08 to 0.36)	NA
Range	0 to 1	NA	0 to 0.86	NA
*Missing, N*	*11*		-	
**Measure of effect**				
RR,* N* (%)	1375 (54.6)	NA	1174 (47.7)	NA
OR,* N* (%)	403 (10.9)	NA	363 (14.8)	NA
HR, *N* (%)	108 (0.5)	NA	106 (4.3)	NA
Mean difference, *N* (%)	941 (34.0)	NA	816 (33.2)	NA
**Relative deviation**^a^				
Median (1. to 3. Quartile)	0.79 (0.33 to 1.41)	NA	0.77 (0.33 to 1.41)	NA
Range	< 0.01 to 11.6		< 0.01 to 11.6	

^a^Three observations were excluded from further analysis because of their extreme relative deviation

*NA* Not applicable

Of the included study results, 368 (13%) were published in journals without an assigned JIF in the respective publication year. Study results from journals with a JIF had more often a high quality of evidence, were less published in open access, and had on average a higher study weight in the meta-analyses and a slightly lower average risk of bias (Table 1). The relative deviation was slightly lower for the “any JIF” group compared to the “no JIF” group (0.98 vs. 1.04, 95% CI −0.15 to 0.04) and not significant (*p* = 0.271).

**Fig. 2:**
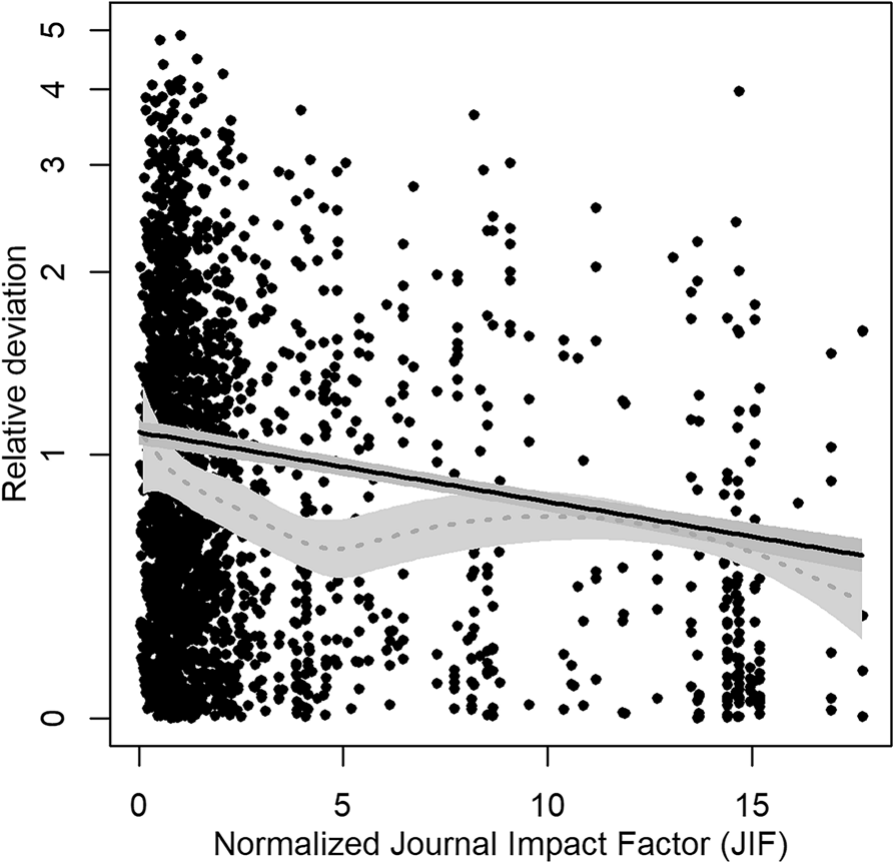
Relative deviation between individual study result and pooled estimate from corresponding meta-analysis depending on the (inflation-corrected) journal impact factor (JIF). The fit of a linear regression and a local polynomial regression (dotted line) together with their 95% confidence bands are drawn. Both regressions are adjusted for the weight that study results received in the meta-analysis, which is fixed at median value for plotting. The regressions are weighted with the JIF to account for heteroscedasticity

From now on, the results refer to the study results with a JIF. The modeled adjusted relationship between JIF and relative deviation, with study weight fixed at its median value, are shown in Fig. 2. For an increase of one JIF, an average decrease in relative deviation of 0.026 (95% CI −0.031 to −0.021) was observed. Compared to an unadjusted model, the effect of JIF was only slightly attenuated by the adjustment for study weight. The steepness of the curve of the local polynomial regression in the beginning indicates that the impact of JIF may be much stronger for inflation-corrected JIFs up to 5, which would correspond to actual JIFs up to 20 in 2018.

The JIF explains only a small proportion of the variability in relative deviations (the multiple *R*^2^ of 6.7% in the linear regression increased by 3.8 percentage points when adding JIF), but is a statistically significant predictor (*F* test: *p* < 0.001). Up to 4.8% of the explanatory power of the JIF could be attributable to the risk of bias.

**Fig. 3:**
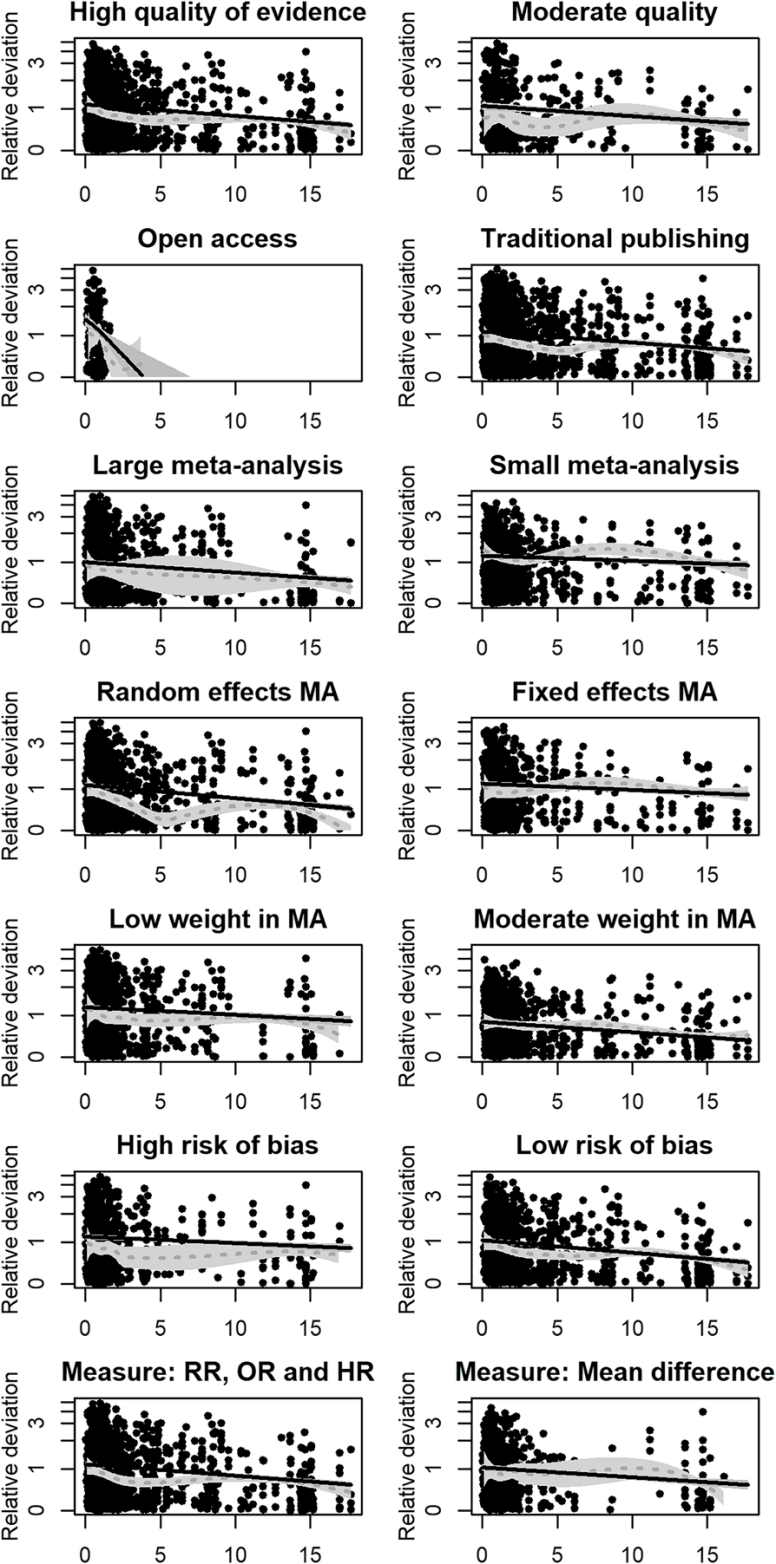
Subgroup analyses of the relationship between relative deviation and journal impact factor (JIF). The fit of a linear regression and a local polynomial regression (dotted line) together with their 95% confidence bands are drawn for each impact factor. All regressions are adjusted for the weight that study results received in the meta-analysis, which is fixed at median value for plotting. Regressions are weighted with the JIF to account for heteroscedasticity

The results from the subgroup analyses are given in Fig. 3. The general pattern of decreasing relative deviation is consistently found in all subgroups, with variations in the form of the nonlinear regression curve. The smallest effect was found in the subgroup of study results from small meta-analyses (−0.013, 95% CI −0.020 to −0.006), the largest for study results from with open access (−0.038, 95% CI −0.576 to −0.181).

**Fig. 4:**
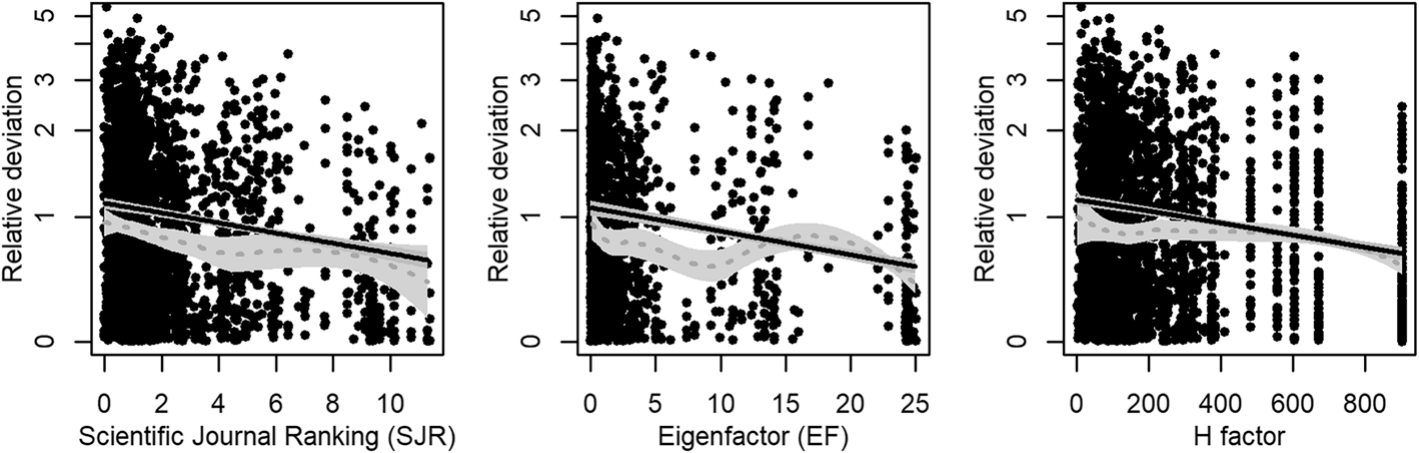
Relationship between relative deviation and other journal impact factors (Scientific Journal Ranking (SJR), Eigenfactor (EF) and H factor). The fit of a linear regression and a local polynomial regression (dotted line) together with their 95% confidence bands are drawn for each impact factor. All regressions are adjusted for the weight that study results received in the meta-analysis, which is fixed at median value for plotting. Regressions are weighted with the respective impact factor to account for heteroscedasticity

The SJR was available for 2599 (91.9%) study results, the EF for 1722 (60.9%) study results and the H-factor for 2743 (97.0%) study results. Also the SJR, the H-factor and the EF showed a decreasing relative deviation with increasing values of the impact measure (Fig. 4).

When an interaction term for study weight and JIF was added to the main linear model, a very small (0.0005) but significant (*p* = 0.012) interaction was found.

## Discussion

### Main findings

We found a positive association between the impact factor of medical journals and the closeness to “truth” of research findings published therein (measured as the relative deviation between the study estimate and the pooled estimate of a meta-analysis as proxy of truth). We used adjusted impact factors to account for inflation. In our sample of medical journals, the inflation-adjusted JIF of 1 roughly translates to an actual JIF of 2.32 in the year 2000 or 4.37 in the year 2018. An increase of the inflation-adjusted JIF by 1 yields an average decrease of the relative deviation by about 0.03. The JIF was found to be a significant predictor, but its predictive power for the accuracy of a study result is very low. It is, therefore, problematic and a practice that should be strongly discouraged to trust a particular study result on the basis of the journal impact; just as it is problematic to infer the impact of an individual study from the journal impact [22].

Is the hypothesized and observed association between the journals’ impact and study findings’ closeness to “truth” a mere reflection of the strengths of well-designed studies? The results from regression analysis suggest that this is not the case. The predictive power of the JIF could be explained by methodological quality only to a small amount (less than 5%). This finding was somewhat surprising initially, as we deemed high methodological standards to be a major journal characteristic to convey the hypothesised advantage of high-rank journals. The low impact we found may, in part, be due to the pragmatic manner by which we assessed the methodological quality. A more elaborate assessment might result in a stronger influence of methodological strength. However, this consequence would affect to which extent the explanatory power of the JIF can be attributed to methodological quality, but it would not affect the predictive power of the JIF as such.

### Strengths and limitations

By putting study results in relation to effect estimates from good-quality Cochrane meta-analyses, we applied a direct and feasible approach to measure the deviation of study results from “truth.” Within this concept, study results with large effect estimates are not suspicious per se, even if the results have not been replicated by more extensive studies. Large effects may occur not only by chance or because of bias due to methodological limitations but also because of (highly) selected study populations or optimized study conditions. These reasons would rather jeopardize the generalizability of the study results than their closeness to “truth.”

The downside of our approach is that the chosen proxy measure for truth is only available for a small proportion of all studies. Studies not meeting the inclusion criteria of Cochrane reviews (i.e., observational studies) and large RCTs, which are excluded due to their inherent correlation with the pooled effect estimate, were not considered. Observational studies, the results of which are less likely to be accurate, might be published rather in lower-impact journals, while large RCTs are published in higher-impact journals. We, therefore, assume that if our study is not representative of the medical literature as a whole, our approach underestimates the association between the JIF and the closeness of study results to the unknown truth.

Our study relies largely on the judgments of review authors regarding the inclusion of studies into the review, the risk of bias assessment and the subsequent valuation of the quality of evidence. These judgments are ultimately at the review authors’ discretion but are not arbitrary, as they are guided by transparent rules. Furthermore, flawed assessments, which are likely to put the validity of specific review findings into question, are less likely to bias our results based on several thousand observations.

### Implications

As the hypothesized association was confirmed, we state that higher-rank journals’ manuscript selection process is effective in identifying research results that are, on average, closer to “truth.” This is the more meritorious as publishing true results may not always be the most promising way to achieve a maximum of citations, a goal appealing to both authors and journal editors. An alternative, potentially incompatible strategy might be to focus on studies in “hot” scientific fields, but the findings of which are supposed to be less likely true [1]. Within a fierce competition for limited publication space, these journals can choose from a larger number of better-quality submissions, and they can use the support of a larger number of experienced and diligent peer reviewers, helping in identifying sound studies and improving the quality of research found worthy to be published [24]. The criteria used lie widely in darkness. Our results suggest they go far beyond the methodological quality as operationalized using the risk of bias assessment. This fits the notion that the peer review process, which critically informs the editorial decision on acceptance, is highly complex and subjective [25]. This is not necessarily a disadvantage. A modeling study showed that exercising some subjectivity in reviewer decisions can be beneficial for the scientific community in processing available information to estimate truth more accurately [26]. We hope that further research into the peer review process sheds light on these aspects.

In all this, we should keep in mind that giving true answers to specific study questions is an essential but not the only function of useful research. False findings caused by intelligent mistakes, unorthodox and immature methods, and diverse samples may broaden the spectrum of scientific thinking more effectively than adding certainty to what is already known to be true.

## Data Availability

The data underlying this article will be shared on reasonable request to the corresponding author.
